# Factors affecting transtemporal window quality in transcranial sonography

**DOI:** 10.1002/brb3.2543

**Published:** 2022-03-03

**Authors:** Lei He, Dong‐Fang Wu, Jing‐Han Zhang, Shuai Zheng, Yi Li, Wen He

**Affiliations:** ^1^ Department of Ultrasound Beijing Tiantan Hospital Capital Medical University Beijing China

**Keywords:** ultrasound, transcranial sonography, transtemporal window

## Abstract

**Objective:**

To assess the influencing factors of transtemporal window quality and identify patients suitable for transcranial sonography (TCS) examination in two‐dimensional imaging.

**Methods:**

In this cross‐sectional study, TCS was performed in 161 consecutive patients through the temporal bone window (TBW) in the neurology or neurosurgery department. Each patient's sex, age, height, weight, and temporal bone thickness (TBT) were collected. After examination, the patients were divided into two groups: TBW success and TBW failure. The data were statistically compared between the two groups.

**Results:**

Among the studied population, the total TBW success rate was 80.1% (95% confidence interval [CI]: 74–86). The TBW success rate was 91.4% (95% CI: 85–98) in males and 70.9% (95% CI: 61–81) in females (*p* = .001). Sex (*p* = .001), age (*p* = .002), height (*p* = .047), and TBT (*p* < .001) showed significant differences between the TBW success and failure groups. In males, only TBT (*p* = .001) showed a significant difference; in females, age (*p* < .001) and TBT (*p* = .003) showed a significant difference. The area under the receiver operating characteristic curve (AUC) of sex, age, and TBT and their combination was 0.686, 0.659, 0.842, and 0.922 (*p* < .001), respectively. The AUC of the combination of parameters was significantly greater than that of age and sex alone (*p* = .007; *p* = .0002) but not greater than that of TBT (*p* = .090).

**Conclusions:**

The TBW success rate varied with sex, age, height, and TBT. Males, younger patients, taller patients, and patients with a thinner temporal bone tended to be more suitable for the examination by TCS.

## Graphical abstract

In this study, we evaluated influencing factors of transtemporal window quality on transcranial sonography.

We found that the thickness of temporal bone was the only influencing factor of transtemporal window quality.

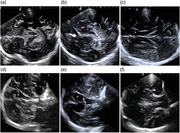

## 1 INTRODUCTION

Transcranial sonography (TCS) has still not become a routine examination in clinical practice, although it has been under development for more than 40 years (Aaslid et al., 1982). In the past few decades, the use of TCS has greatly increased; TCS can be applied in a wide range of settings, including in general clinical practice, neuro‐intensive and general intensive care units, operating rooms, and emergency departments. TCS has been widely applied in many hospitals for assessing intracranial vessels (Baumgartner, 2003; Nedelmann et al., 2009) and abnormality of parenchyma. The main reason TCS is limited in each application is the failure to obtain transtemporal acoustic windows in a certain population due to the limited penetration of skull by the ultrasound beam. There are four main acoustic windows used in clinical practice: the transtemporal, transorbital, transfrontal, and transoccipital bone windows (Robba et al., 2019). Generally, the temporal bone window (TBW) is the most common acoustic window and has become the standard window used for TCS examination in adults. However, the rate (5%–23%; Hennerici et al., 1987; Itoh et al., 1993) and influencing factors of the TBW success vary in different studies. Probably, one reason was the selection bias of the population as most studies focused on certain disease, such as stroke patients (Kollár et al., 2004; Kwon et al., 2006; Schreuder et al., 2009; Bazan et al., 2015). Different races may be the second reason. Bazan et al. (2015) showed that the incidence of TBW success was lower among African compared with White individuals. The equipment for assessing the determinants of TBW success was transcranial Doppler (TCD) in most studies, and the definition of success was the flow signals of cerebral artery—usually the middle cerebral artery—could be measured (Halsey, 1990; Yagita et al., 1996; Kwon et al., 2006; Lee et al., 2020; Brisson, Santos, et al., 2021; Schreuder et al., 2009). Few literature researched the influencing factors of the appearance of cerebral parenchyma by bidimensional ultrasound of TCS. The main purpose of the present study was to assess the main factors that influence TBW success and identify patients who are suitable for TCS examination in Chinese population.

## 2 METHODS

### 2.1 Study population

This was a cross‐sectional study conducted in a single institution. The privacy rights of the human subjects were always observed. This study was approved by the Research Ethics Board of Beijing Tiantan Hospital, Capital Medical University.

The study population comprised consenting patients who presented to the neurology or neurosurgery department of the Beijing Tiantan Hospital, as these patients may be more likely to undergo brain examinations.

The inclusion criterion was as follows: (1) age greater than 18 years.

The exclusion criteria were as follows: (1) loss of integrity of the skull or soft tissue of the temple, such as in cases of local bone fracture or soft tissue infection; (2) any brain surgery or other craniocerebral treatment, such as radiotherapy and chemotherapy of the brain; and (3) refusal to participate in this study.

### 2.2 Data collection

After enrollment, the clinical characteristics of each patient were recorded, including demographics (sex, age, height, and weight) and cranial computed tomography (CT), which was used to measure the temporal bone thickness (TBT) in the axial image slices between the midbrain and the lower basal ganglia.

### 2.3 Ultrasound examination techniques

All patients underwent transcranial ultrasound examination by a phased‐array ultrasound system equipped with a 1.6–2.5 MHz and 5–12 MHz (measure temporal soft tissue) transducer (EPIQ 7; Philips, USA). A dynamic range of 45–55 dB and a penetration depth of 14–16 cm through the transtemporal bone window were used (Walter & Školoudík, 2014). The image brightness and time gain compensations were adapted as needed for each patient.

### 2.4 TCS protocol

**FIGURE 1:**
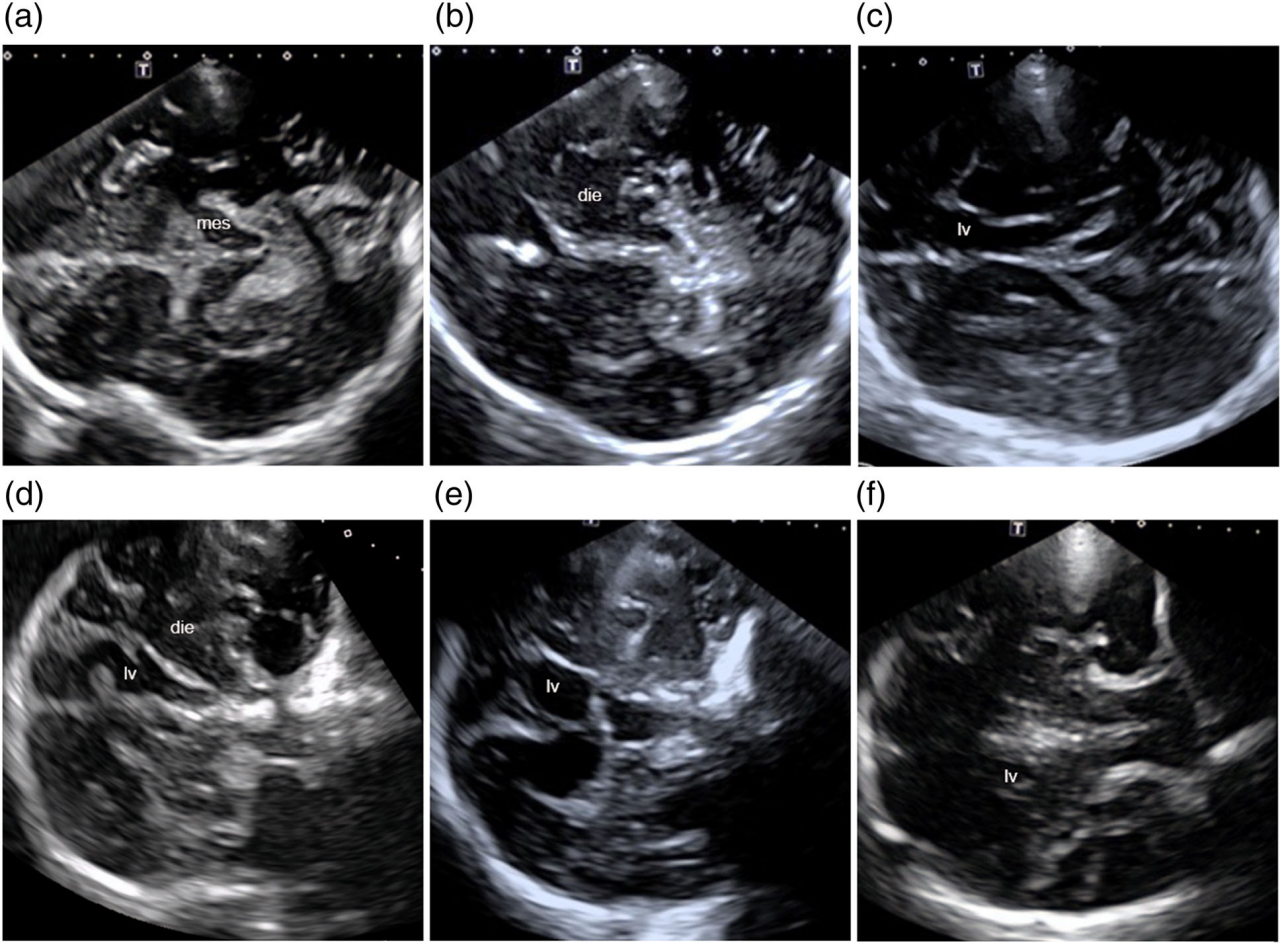
lustration of temporal bone window (TBW) success: (1) on the axial plane, the mesencephalic(mes) (a), diencephalic(die) (b), and ventricular(lv) (c) regions are displayed from inferior to superior; (2) on the coronal plane, the anterior horn (d), body (e), and inferior horn of the lateral ventricle (f) are displayed from anterior to posterior

For all patients, the first step of the TCS examination was standardized imaging on the axial plane as recommended (Walter & Školoudík, 2014) through the TBW (with the marker toward the eye through either the left or right TBW). Then, the transducer was turned 90° clockwise for imaging on the coronal plane. The criteria of successful imaging through the TBW (TBW success) were defined according to the literature (Walter & Školoudík, 2014; Mäurer et al., 1998) as follows: (1) on the axial plane, the mesencephalic, diencephalic, and ventricular regions were observed from inferior to superior; (2) on the coronal plane, the anterior horn, body, and inferior horn of the lateral ventricle were observed from anterior to posterior (Figure 1). The examination was conducted by two experienced sonographers to ensure that the best images were obtained.

### 2.5 Statistical analysis

The study population was first described, and then the consistency of the evaluation of quantitative paired variables was assessed using the intraclass correlation efficient (ICC). The characteristics of the TBW failure and TBW success groups were compared. Qualitative variables are expressed as numbers (percentages) and were tested using the *χ* ^2^ test. For quantitative variables, the normality of the distribution was tested using the Shapiro–Wilk test, and these data are expressed as the mean (SD) or median (interquartile range [IQR]). Quantitative data were compared using Student's *t‐*test when they followed a normal distribution; otherwise, they were compared by the Mann–Whitney test.

TBW success is described as a percentage with a 95% confidence interval (CI). As the TBW success rate has been reported to range from 40% to 90% in the literature, we chose a TBW success rate of approximately 65%. A sample size of 114 subjects would have approximately 90% power to determine the estimated success rate using a 95% CI with ±8% accuracy and considering an alpha risk of 0.05. Risk factors for TBW failure were also investigated by the two‐sided *Z*‐test with pooled variance. The significance level of the test was 0.05.

A multivariate analysis with logistic regression was used to identify independent predictors of TBW success, with a *p*‐value ≤.10 for entry into the model and a *p*‐value >.10 for removal. SPSS (IBM^®^ SPSS Statistics version 26) statistical software was used for all data analyses.

Receiver operating characteristic (ROC) curve analysis for TBW success was performed using MedCalc software (MedCalc version 19.6.1) and a comparison of the area under the ROC curve (AUC) of different predictors was performed.

## 3 RESULTS

### 3.1 Study population

A total of 161 patients were included in the present study between October 2020 and February 2021. We excluded five patients with only one side TBW success. Finally, the study included 86 females (55%) and 70 males (45%), with a mean age of 53 ± 11 (20–80) years. The demographic data of the patients are summarized in Table 1.

**TABLE 1:** Demographic and clinical characteristics of patients

Variables	*n* = 156
Gender (f)	86 (55.1%)
Age, year	53 ± 11
Height, cm	165 ± 7
Weight, kg	66 ± 12
BMI, kg/m^2^	24 ± 3
TST, cm	0.56 ± 0.12
TBT, cm	0.27 ± 0.06

*Note*: Continuous values are given as the mean ± SD (minimum‐maximum). No. of females (%)/males (%)

Abbreviations: BMI, body mass index; TBT, temporal bone thickness; TST, temporal soft tissue thickness.

### 3.2 Agreement between paired variables

The ICC of the thickness of the temporal soft tissue (TST) and TBT between the right and left sides was 0.918 (*p <* .001) and 0.932 (*p* < .001) (average measures), respectively. The consistency of the above parameters showed a significant agreement. We used the mean values of paired parameters for statistical analysis of the data. The interobserver agreement on TBW success was excellent (*κ* = 0.95, *p* < .001).

### 3.3 TBW success rate

The total TBW success rate was 80.1% (95% CI: 74–86). The TBW success rate in males and females was 91.4% (95% CI: 85–98) and 70.9% (95% CI: 61–81), respectively (*p =* .001).

### 3.4 Differences in parameters between TBW success and failure

Sex (80% vs. 19.9%, *p =* .001), age (52 vs. 59, *p =* .002), height (166 vs. 163, *p* = .047), and TBT (0.26 vs. 0.33, *p* < .001) showed a significant difference between the TBW success and failure groups, while weight (66 vs. 66, *p* = .953), body mass index (BMI) (23.9 vs. 24.9, *p* = .166), and TST (0.56 vs. 0.56, *p* = .962) did not show a significant difference. A comparison of the parameters between the TBW success and failure groups is summarized in Table 2.

**TABLE 2:** Comparison of variables between temporal bone thickness (TBW) success and failure

Variables	TBW success	TBW failure	Value	*p*
Sex	125 (80.1%)	31 (19.9%)	10.183	.001
Male	64 (91.4%)	6 (8.6%)		
Female	61 (70.9)	25 (29.1%)		
Age	52 ± 11	59 ± 9	3.148	.002
Height	166 ± 7	163 ± 9	−2.006	.047
Weight	66 ± 11	66 ± 13	0.59	.953
BMI	23.9 ± 3.2	24.9 ± 3.5	1.392	.166
TST*	0.56 ± 0.13	0.56 ± 0.10	0.48	.962
TBT^#^	0.26 ± 0.05	0.33 ± 0.06	4.839	<.001

Abbreviation: BMI, body mass index.

* Thickness of temporal soft tissue.

^#^ thickness of temporal bone.

When the data were divided by sex, in males, only TBT (0.25 vs. 0.38, *p* = .001) showed a significant difference. In females, age (48 vs. 60, *p* < .001) and TBT (0.27 vs. 0.33, *p* = .003) showed a significant difference. Although, TBT (0.38 vs. 0.33, *p* = .826) had no significant difference between males and females in TBW failure. A comparison of these data is shown in Table 3.

**TABLE 3:** Comparison of parameters between temporal bone window (TBW) success and failure split by sex

Variables	Sex	TBW success	TBW failure	Value	*p*
Age	M	55 ± 10	51 ± 12	−0.772	*p =* .443
	F	48 ± 12	60 ± 8	4.627	*p <* .001
Height	M	171 ± 5	173 ± 8	0.788	*p* = .433
	F	161 ± 5	160 ± 7	−0.33	*p =* .742
Weight	M	72 ± 11	83 ± 13	2.284	*p =* .26
	F	60 ± 8	62 ± 10	0.894	*p =* .374
BMI	M	24.6 ± 3.3	27.7 ± 2.0	2.246	*p =* .28
	F	23.2 ± 3.0	24.1 ± 3.5	1.165	*p =* .248
TST*	M	0.59 ± 0.14	0.55 ± 0.13	−0.708	*p =* .482
	F	0.53 ± 0.12	0.56 ± 0.09	1.314	*p* = .193
TBT^#^	M	0.25 ± 0.05	0.38 ± 0.05	3.787	*p =* .001
	F	0.27 ± 0.5	0.33 ± 0.6	3.13	*p* = .003

Abbreviation: BMI, body mass index.

* Thickness of temporal soft tissue.

^#^ Thickness of temporal bone.

**FIGURE 2:**
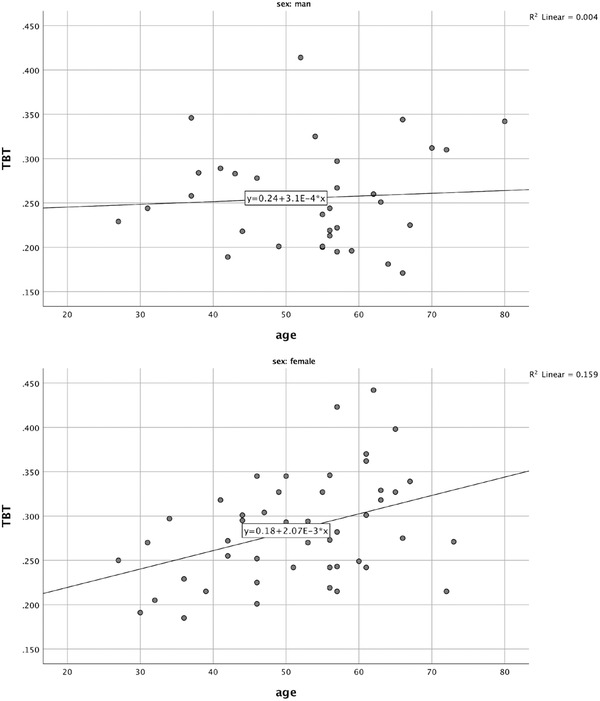
Correlation between age and temporal bone thickness in females and males

A positive linear correlation between age and TBT (*r* = 0.398; *p* = .005) was found in females, while no linear correlation (*r* = 0.066; *p =* .712) was found in males (Figure 2).

### 3.5 Multivariate predictors of acute TBW success

Multivariate analysis revealed that age (odds ratio [OR] = 0.05, 95% CI 0.01–0.09, *p* = .040), sex (male; OR = 11.74, 95% CI 9.78–15.12, *p* = .026), and TBT (OR = 0.71, 95% CI 0.45–0.93, *p* = .009) significantly affected TBW success.

**FIGURE 3:**
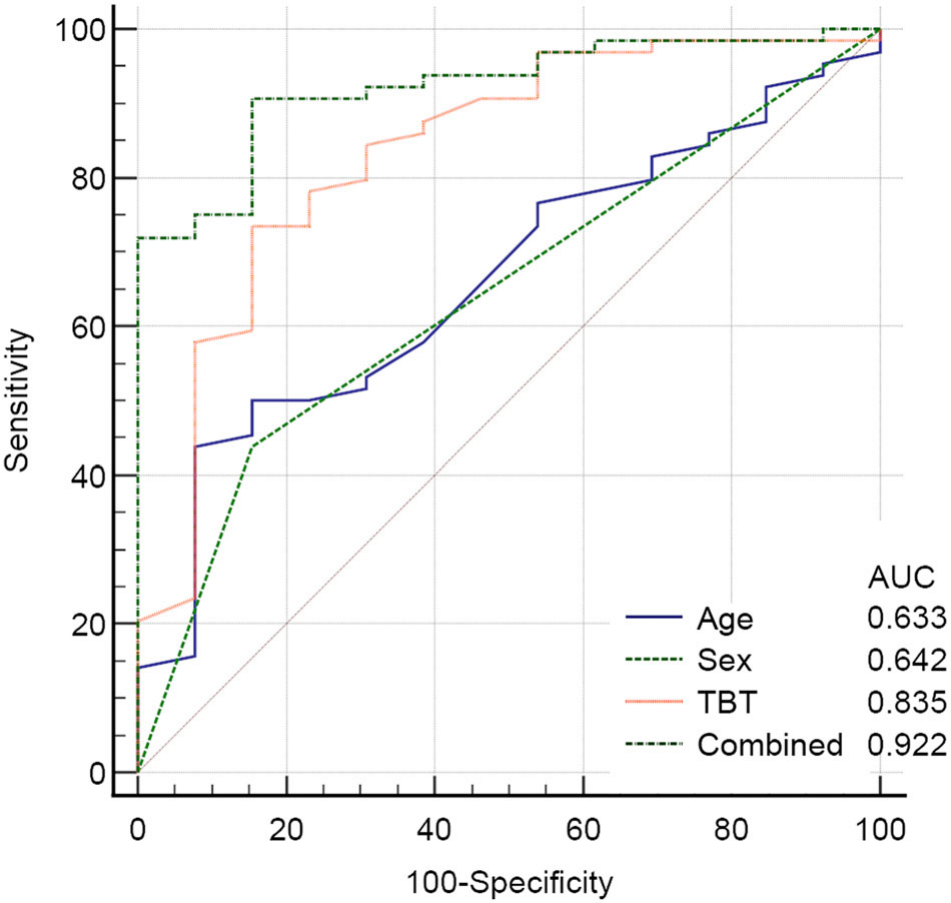
Receiver operating characteristic curves of the combination of predictors versus age, sex, and TBT for the prediction of temporal bone window (TBW) success. The result of the combination of predictors was superior to that of age and sex, with a significantly greater area under the curve (0.922 vs. 0.663; *p* = .007; .922 vs. .642; *p* = .0002)

ROC curves were created to analyze the diagnostic value of sex, age, TBT, and their combination in predicting TBW success. The AUC of sex, age, TBT, and their combination was 0.686 (*p <* .001), 0.659 (*p* < .001), 0.842 (*p <* .001), and 0.922 (*p* < .001), respectively. A comparison of the combination of predictors versus sex, age, and TBT is shown in Figure 3. The AUC of the combination of parameters was significantly greater than that of age and sex alone (0.922 vs. 0.663 *p* = .007; 0.922 vs. 0.642 *p* = .0002) but was not greater than that of TBT (0.922 vs. 0.835 *p =* .090).

## 4 DISCUSSION

The results of this study showed that age, sex, and TBT significantly influenced TBW success, while height, weight, BMI and TST did not significantly influence TBW success, which was basic consistency with the results reported in most literatures (Brisson, Santos, et al., 2021; Brunser et al., 2012; Bazan et al., 2015; Jarquin‐Valdivia et al., 2004; Kwon et al., 2006; Lee et al., 2020;). There were other variables we did not include in this study, such as the homogeneity or density of bone, hypertension, smoking, and cholesterol. These factors either were inconsistency among literatures or made the issue complex.

One of the differences in our study was the definition of TBW success. Although assessing the artery flow was the important utility of transcranial sonography, evaluating abnormal echo in the parenchyma was also a main application area. The intracranial structures that need to be observed vary among diseases. The use of TCS in evaluating movement disorders may be the most widespread application of TCS. The finding of an increase in the echogenicity of the substantia nigra has enabled the reliable diagnosis of Parkinson's disease (PD), with a high predictive value (Berg et al., 2008). Cerebrovascular disease as a comorbidity in PD patients may adversely affect clinical outcome. Apart from bidimensional mode ultrasound, transcranial color‐coded sonography has already been reported in patients with PD. The changes on cerebral hemodynamics may determine the severity and clinical outcomes of PD (Brisson, de Cássia Leite Fernandes, et al., 2021). Standardized axial imaging of the butterfly‐shaped midbrain is performed with the transducer placed at the preauricular site and the patient in a supine position. Generally, no other scanning planes are needed when TCS is performed for the diagnosis of PD. On the area of sonothrombolysis, the efficacy may also be influenced by poor acoustic window (Novotny et al., 2018). Poor acoustic window means ultrasound beam was scattered and absorption, then subsequently reduced passage of the ultrasound beam. When TCS is performed in the context of other diseases, such as intracranial hemorrhage and brain tumors, a greater acoustic area bone window is needed (Becker et al., 1991; Camps‐Renom et al., 2017). Although approximately 70% cerebral hemorrhages occur in the ganglia, the cerebral lobes are also common sites (Chen et al., 2018). This means that more brain structures need to be shown to minimize missed diagnoses. Therefore, we defined TBW success and failure as described above.

The total TBW success rate in the study was 80.1%, which is lower than that reported in some literature (Bogdahn et al., 1990) and potentially due to the stricter standards we defined. The TBW success rate tended to be higher among males than females, which is consistent with the literature (Becker et al., 1992; Oliveira et al., 2017; Niesen et al., 2018; Stolz et al., 2002; Tao et al., 2019). The age difference between the TBW success and failure groups had not statistical significance in man (55 vs. 51). However, it had a statistical significance between females (48 vs. 60), which period coincided with the period of menopause (Ginsberg, 1991). Theoretically, as the level of estrogen declines, the characteristics of bone also change; for example, the bone density decreases, which manifests as osteoporosis. Osteoporosis not only reduces the density of the skull but also, more importantly, makes the bone inhomogeneous, which can result in the formation of many ultrasound‐reflective interfaces, making it more difficult for sound waves to penetrate the skull (Serpe & Rho, 1996; Wear, 2020). Lee et al. (2020) and Schreuder et al. (2009) demonstrated that the density or inhomogeneity of bone was influencing factors for TBW success, while Kollár et al. (2004) showed that density values were similar when comparing different image quality groups. In our study, age was positively linearly correlated with TBT in females, which means TBT increasing with age. TBT was acknowledged for influencing TBW success in literatures, which was also demonstrated in our study. These could explain the high TBW failure in old females, and the age coincided with the period of menopause may be just a coincidence.

There was also a significant difference in height (166 vs. 163, *p =* .047) in the total population but not in males or females when the data were split by sex. This inconsistency might be ascribed to the few cases of TBW failure among males (6/70). Thus, it may be inappropriate to make the conclusion that height was not significantly different between the two groups.

TBT was the most important factor of TBW success, as demonstrated by comparison of the AUC: the AUC of the combination of parameters was not significantly greater than that of TBT (0.922 vs. 0.835 *p* = .090) but was significantly greater than that of age and sex alone (0.922 vs. 0.663 *p* = .007; 0.922 vs. 0.642 *p* = .0002).

## 5 CONCLUSIONS

In this study, there is a significant difference in the display rate between males and females, with males being more suitable for TCS examination regardless of age, as well as females younger than 48 years. The temporal bone tended to be thicker as females get older, while it did not change in man. TBT is the only factor, while weight, BMI, and TST are not factors that could influence TBW success or failure.

## CONFLICT OF INTEREST

The authors declare no conflict of interest.

## FUNDING INFORMATION

National Natural Science Foundation of China (ID 81730050).

### PEER REVIEW

The peer review history for this article is available at https://publons.com/publon/10.1002/brb3.2543.

## Data Availability

The original contributions presented in the study are included in the article. Further inquiries can be directed to the corresponding author.
